# FPLS-DC: functional partial least squares through distance covariance for imaging genetics

**DOI:** 10.1093/bioinformatics/btae173

**Published:** 2024-03-29

**Authors:** Wenliang Pan, Yue Shan, Chuang Li, Shuai Huang, Tengfei Li, Yun Li, Hongtu Zhu

**Affiliations:** Academy of Mathematics and Systems Science, Chinese Academy of Sciences, Beijing 100190, China; Departments of Biostatistics, Statistics, Genetics, and Computer Science and Biomedical Research Imaging Center, University of North Carolina, Chapel Hill, NC 27599, USA; Department of Statistical Science, School of Mathematics, Sun Yat-sen University, Guangzhou 510275, China; Departments of Biostatistics, Statistics, Genetics, and Computer Science and Biomedical Research Imaging Center, University of North Carolina, Chapel Hill, NC 27599, USA; Departments of Radiology and Biomedical Research Imaging Center, University of North Carolina, Chapel Hill, NC 27599, USA; Departments of Biostatistics, Statistics, Genetics, and Computer Science and Biomedical Research Imaging Center, University of North Carolina, Chapel Hill, NC 27599, USA; Departments of Biostatistics, Statistics, Genetics, and Computer Science and Biomedical Research Imaging Center, University of North Carolina, Chapel Hill, NC 27599, USA; Departments of Radiology and Biomedical Research Imaging Center, University of North Carolina, Chapel Hill, NC 27599, USA

## Abstract

**Motivation:**

Imaging genetics integrates imaging and genetic techniques to examine how genetic variations influence the function and structure of organs like the brain or heart, providing insights into their impact on behavior and disease phenotypes. The use of organ-wide imaging endophenotypes has increasingly been used to identify potential genes associated with complex disorders. However, analyzing organ-wide imaging data alongside genetic data presents two significant challenges: high dimensionality and complex relationships. To address these challenges, we propose a novel, nonlinear inference framework designed to partially mitigate these issues.

**Results:**

We propose a functional partial least squares through distance covariance (FPLS-DC) framework for efficient genome wide analyses of imaging phenotypes. It consists of two components. The first component utilizes the FPLS-derived base functions to reduce image dimensionality while screening genetic markers. The second component maximizes the distance correlation between genetic markers and projected imaging data, which is a linear combination of the FPLS-basis functions, using simulated annealing algorithm. In addition, we proposed an iterative FPLS-DC method based on FPLS-DC framework, which effectively overcomes the influence of inter-gene correlation on inference analysis. We efficiently approximate the null distribution of test statistics using a gamma approximation. Compared to existing methods, FPLS-DC offers computational and statistical efficiency for handling large-scale imaging genetics. In real-world applications, our method successfully detected genetic variants associated with the hippocampus, demonstrating its value as a statistical toolbox for imaging genetic studies.

**Availability and implementation:**

The FPLS-DC method we propose opens up new research avenues and offers valuable insights for analyzing functional and high-dimensional data. In addition, it serves as a useful tool for scientific analysis in practical applications within the field of imaging genetics research. The R package FPLS-DC is available in Github: https://github.com/BIG-S2/FPLSDC.

## 1 Introduction

With the advancement of imaging and genetic technologies, there has been a surge in large-scale biomedical studies like the UK Biobank (UKB) study (Miller *et al.* 2016). These studies have amassed a wide array of data spanning imaging, genetics, health factors, and electronic health records. Among this vast spectrum of information, organ-specific imaging traits have emerged as essential biomarkers for understanding organ development, aging, and the diagnosis and prognosis of diseases (Zhou *et al.* 2021). Furthermore, a joint analysis of comprehensive organ-wide imaging and genetic data not only aids in deciphering the genetic architectures behind organ structure and function (Bearden and Thompson 2017, Elliott *et al.* 2018, Zhao *et al.* 2021, 2023), but also helps in detecting pertinent genetic markers associated with various organ-related disorders, like Osteoarthritis (Le and Stein 2019, Wilkinson and Zeggini 2021). Over time, this extensive collection of data could pave the way for mapping potential biological pathways that link genetics to imaging endophenotypes for different organs, such as the brain and heart, and relate these to clinical outcomes that are confounded with health factors.Nevertheless, the joint analysis of imaging and genetic data poses considerable challenges to existing statistical methods (Liu and Calhoun 2014, Shen and Thompson 2020, Zhu *et al.* 2023). As elucidated by Vounou *et al.* (2010), methods used in imaging genetics can be classified into four categories: candidate phenotype-candidate gene association (CPCGWA), candidate phenotype-genome-wide association (CPGWA), brain-wide candidate gene association (BWCGA), and brain-wide genome-wide association (BWGWA). These categories are distinguished by the dimensionality of the genotypes and imaging phenotypes involved. Our primary research interest lies in addressing several major computational and methodological challenges related to BWCGA and BWGWA. These challenges primarily stem from the high dimensionality of genetic and imaging data, along with the intricate spatial structures inherent to them.

A large body of literature exists on the development of statistical methods for BWGWA and BWCGA (Liu and Calhoun 2014, Shen and Thompson 2020). Mass-univariate linear modeling (MULM) is a commonly utilized method for detecting linear genotype–phenotype relationships (Stein *et al.* 2010, Hibar *et al.* 2011, Huang *et al.* 2015). However, MULM involves a substantial number of comparisons, which may limit the power to detect even moderate signals. To partially mitigate this problem, various multivariate approaches, such as partial least squares correlation and canonical correlation analysis, have been introduced to identify multivariate linear genotype–phenotype associations (Liu and Calhoun 2014, Grellmann *et al.* 2015). Moreover, regularization methods have been used to handle high-dimensional scenarios, with the goal of selecting a small number of features (Vounou *et al.* 2010, Kohannim *et al.* 2012, Yang *et al.* 2015). Recently, Huang *et al.* (2017) proposed a functional genome-wide association analysis (FGWAS) framework designed to detect linear genotype–phenotype relationships, while accounting for functional features, such as functional smoothness and correlation, inherent in imaging data. Lastly, there is a growing interest in delineating nonlinear genotype–phenotype relationships. This has been pursued by using distance correlation metrics for random vectors (Székely *et al.* 2007, Székely and Rizzo 2013), including the greedy projected distance correlation [G-PDC, Fang *et al.* (2018)] and weighted distance correlation [wdCor, Wen *et al.* (2020)] methods.

In this article, we propose a functional partial least squares through distance covariance (FPLS-DC) framework. Our goal is to delineate nonlinear genotype–phenotype relationships while explicitly accounting for the functional features present in imaging data. Our FPLS-DC framework consists of three main steps. First, we utilize an alternative partial least squares (APLS) approach (Delaigle and Hall 2012) to extract a low-dimensional informative projection vector (IPV) from the imaging data. Next, we use simulated annealing algorithm to maximize the distance covariance between genetic markers and the IPV. Finally, we implement a rapid testing procedure for conducting BWCGA and BWGWA efficiently. To examine the finite sample properties of our FPLS-DC, we carry out extensive simulations and real data analyses. Moreover, to address the challenge posed by correlation between predictors, we introduce the iterative FPLS-DC (I-FPLS-DC) algorithm. This iterative approach further amplifies the analytical capabilities of our methodology.

The structure of the remainder of the paper is as follows. Section 2 lays the groundwork by providing necessary preliminary information for the subsequent discussions. In Section 3, we provide a detailed description of the FPLS-DC and I-FPLS-DC algorithms. Section 4 presents the outcomes of our Monte Carlo simulations. In Section 5, we carry out a large-scale real data analysis using UKB. Finally, Section 6 discusses potential extensions of the proposed algorithms and outlines possible directions for future work.

## 2 Preliminary

In this section, we introduce two fundamental components of the FPLS-DC and I-FPLS-DC algorithms: the APLS algorithm and distance covariance.

### 2.1 APLS algorithm

The functional linear model (FLM) is a statistical framework designed to model the relationship between a scalar response variable *V* and a functional predictor variable U(s), the latter variable is defined on a nondegenerate and compact set S, satisfying $\int_{S}E\{U^{2}(s)\}ds<\infty$. In this framework, the response variable is hypothesized to be a function of the predictor variables, and the relationship is modeled as a linear combination of basis functions of the predictor variable:

(1) $$\begin{array}{c} V=a_{0}+\int_{S}U(s)b(s)ds+\varepsilon , \end{array}$$

where $a_{0}$ is a scalar parameter, ε is a scalar random variable with E(ε|U)=0, and b(s) is an unknown coefficient function on S.

The partial least squares (PLS) algorithm can be used in FLM to find a series of orthogonal basis functions ${\left\{\psi_{j}(s)\right\}}_{j\geq 1}$ to approximate the functional coefficient b(s). However, the computational implementation of PLS can prove challenging, particularly in the context of high-dimensional and complex functional data. To address these limitations in terms of computational efficiency and stability, the APLS approach was designed. Specifically, APLS replaces the singular value decomposition (SVD) used in PLS with an eigenvalue decomposition, which may be more efficient for specific types of data.

The APLS algorithm initiates with a covariance function K(s,t)=Cov(U(s),U(t)). The algorithm then leverages an iterative process to compute a sequence of nonorthogonal basis functions $\psi_{1}(s),\ldots ,\psi_{q}(s)$, which are subsequently used to approximate the functional coefficient b(s). Each basis function is defined relative to the preceding ones, using a recursion formula that incorporates the covariance function K(s,t). Specifically, we calculate

$$\begin{array}{c} \psi_{j}(s)=\{\begin{array}{cc} \int_{S}K(s,t)b(t)dt & \text{for}\;j=1,\\ \int_{S}\psi_{j-1}(t)K(s,t)dt, & \text{for}\;j=2,\ldots ,q. \end{array} \end{array}$$

Let $\gamma ={\left(\gamma_{1},\ldots ,\gamma_{q}\right)}^{T}$. The approximation of b(s) is given by $\sum_{j=1}^{q}\gamma_{j}\psi_{j}(s)$, where the coefficients $\gamma_{j}$ are chosen to minimize the mean squared error between *V* and U(s) based on the basis functions. Consequently, the prediction of *V* is approximated by estimating the conditional expectation:

$$\begin{array}{c} E\{V|U(s)\}=E(V)+\sum_{j=1}^{q}\gamma_{j}\int_{S}\{U(s)-EU(s)\}\psi_{j}(s)ds. \end{array}$$

Given the random samples $(U,V)={\left\{(U_{i}(s),V_{i})\right\}}_{i=1,\ldots ,n}$ from (U(s),V), the estimation of E(V|U(s)) proceeds as follows:

**Step 1:** Estimate $\left\{{\hat{\psi }}_{1}(s),\ldots ,{\hat{\psi }}_{q}(s)\right\}$ by

(2) $${\hat{\psi }}_{j}(s)=\{\begin{array}{cc} n^{-1}\sum_{i=1}^{n}\left(V_{i}-\bar{V}\right)\{U_{i}(s)-\bar{U}(s)\}, & \text{for}\;j=1;\\ \int_{S}{\hat{\psi }}_{j-1}(t)\hat{K}(s,t)dt, & \text{for}\;j=2,\ldots ,q, \end{array}$$

where $\hat{K}(s,t)=n^{-1}\sum_{i=1}^{n}\{U_{i}(s)-\bar{U}(s)\}\{U_{i}(t)-\bar{U}(t)\}$, $\bar{U}(s)=n^{-1}\sum_{i=1}^{n}U_{i}(s)$, and $\bar{V}=n^{-1}\sum_{i=1}^{n}V_{i}$.

**Step 2:** Estimate $\hat{\gamma }={\left({\hat{\gamma }}_{1},\ldots ,{\hat{\gamma }}_{q}\right)}^{T}$ to minimize

(3) $$\frac{1}{n}\sum_{i=1}^{n}[(V_{i}-\bar{V})-\sum_{j=1}^{q}\gamma_{j}\int_{S}\{U_{i}(s)-\bar{U}(s)\}{\hat{\psi }}_{j}(s)ds{]}^{2}.$$

**Step 3:** Estimate the prediction function $E\{V_{i}|U_{i}(s)\}$ by

$$\begin{array}{c} \bar{V}+\sum_{j=1}^{q}{\hat{\gamma }}_{j}\int_{S}\{U_{i}(s)-\bar{U}(s)\}{\hat{\psi }}_{j}(s)ds. \end{array}$$

The APLS algorithm has certain limitations that need to be considered. Firstly, it may not be suitable when the functional predictor variable exhibits a complex dependency structure, such as nonstationarity or nonlinearity. This is because the algorithm assumes a linear relationship between the predictor and the response variable, which may not be valid in these cases. In addition, APLS might not perform optimally with datasets having a low signal-to-noise ratio or with highly correlated predictors.

### 2.2 Distance covariance

Distance covariance (dcov) is a statistical measure used to determine the degree of dependence between two random vectors, $W\in R^{p}$ and $V\in R^{q}$. Initially introduced by Székely *et al.* (2007), it measures the strength of the relationship between two random vectors. Let’s proceed with the formal definition of distance covariance and its empirical unbiased variant.

Definition 2.1. *The distance covariance (dcov) between random vectors W and V with finite first moments is the nonnegative number* dcov(W,V)*defined by*

$$\begin{array}{c} {\text{dcov}}^{2}(W,V)={\left(c_{p}c_{q}\right)}^{-1}\int_{R^{p+q}}\frac{{\left|\phi_{W,V}(t,s)-\phi_{W}(t)\phi_{V}(s)\right|}^{2}}{|t{|}_{p}^{1+p}|s{|}_{q}^{1+q}}d\mathrm{tds} \end{array}$$

*where* $\phi_{W,V}$, $|t{|}_{p}$*and* $|s{|}_{q}$*are, respectively, the Euclidean norms of* $t\in R^{p}$*and* $s\in R^{q}$, $\phi_{W}$*and* $\phi_{V}$*are the characteristic functions of* (W,V)*, W and V, respectively, and* $c_{d}=\pi^{(1+d)/2}/\Gamma ((1+d)/2)$.

Let ${\left(W_{i},V_{i}\right)}_{i=1,2,3}$ be i.i.d. with (W,V) and there exists an equivalent form of distance covariance that involves absolute differences of *W* and *V*. Specifically, the distance covariance between *W* and *V* can be expressed as:

$$\begin{array}{c} {\text{dcov}}^{2}(W,V)=E\left[{\left|W_{1}-W_{2}\right|}_{p}{\left|V_{1}-V_{2}\right|}_{q}\right]+E{\left|W_{1}-W_{2}\right|}_{p}E{\left|V_{1}-V_{2}\right|}_{q}\\ -2E\left[{\left|W_{1}-W_{2}\right|}_{p}{\left|V_{1}-V_{3}\right|}_{q}\right]. \end{array}$$

Empirical unbiased versions of distance covariance can be obtained from finite samples of *W* and *V* by substituting the expected values with sample means.

Definition 2.2. *Let* $\left\{(W_{i},V_{i}),i=1,\ldots ,n\right\}$*denote a sample of observations from the joint distribution* (W,V)*of random vectors W and V. Let* $A=(a_{ij})$*be the Euclidean distance matrix of the sample* $W_{1},\ldots ,W_{n}$*and* $B=(b_{ij})$*be the Euclidean distance matrix of the sample* $V_{1},\ldots ,V_{n}$*. Then if E(*|W|+|V|)<∞*, for* n>3*, then* ${\text{dcov}}_{n}^{2}(W,V)={\left\{n(n-3)\right\}}^{-1}\sum_{i\neq j}{\tilde{A}}_{ij}{\tilde{B}}_{ij}$*is an unbiased estimator of squared population distance covariance* ${\text{dcov}}^{2}(W,V)$*, where*

$$\begin{array}{c} {\tilde{A}}_{ij}=\{\begin{array}{cc} a_{ij}-{\bar{A}}_{il}-{\bar{A}}_{kj}+{\bar{A}}_{kl}, & i\neq j,\\ 0, & i=j, \end{array} \end{array}$$

*in which* ${\bar{A}}_{il}={\left(n-2\right)}^{-1}\sum_{\ell =1}^{n}a_{i\ell }$, ${\bar{A}}_{kj}={\left(n-2\right)}^{-1}\sum_{k=1}^{n}a_{kj}$*, and* ${\bar{A}}_{kl}={\left\{(n-1)(n-2)\right\}}^{-1}\sum_{k,\ell =1}^{n}a_{k\ell }$*, and the form of* ${\tilde{B}}_{ij}$*is similar to that of* ${\tilde{A}}_{ij}$.

The distance covariance has shown promising results in detecting both linear and nonlinear relationships between random vectors.

## 3 Materials and methods

In this section, we introduce the FPLS-DC algorithm and the I-FPLS-DC algorithm. We first establish some essential notations to facilitate understanding of the content to follow. Let $X={\left(X_{1},\ldots ,X_{p}\right)}^{T}$ be a p×1 random vector, and Y(s) be a random functional response variable defined on S. We denote $(X,Y(s))=(X_{1},\ldots ,X_{p},Y(s))={\left\{(X_{i1},\ldots ,X_{ip},Y_{i}(s))\right\}}_{i=1,\ldots ,n}$ as a random sample from (X,Y(s)). For $\beta ={\left(\beta_{1},\ldots ,\beta_{p}\right)}^{T}$, we define the zero norm $\left||\beta |{|}_{0}=\sum_{j=1}^{p}I(\beta_{j}\neq 0\right)$ and the *p*-norm $||\beta |{|}_{p}={\left(\sum_{j=1}^{q}|\beta_{j}{|}^{p}\right)}^{1/p}$ for p∈[1,∞), where I(A) is an indicator function of an event *A*. For any index set A⊂{1,…,p}, we denote |A| as its cardinality and define $X_{A}={\left(X_{ij},j\in A\right)}^{T}\in R^{|A|\times n}$.

### 3.1 FPLS-DC algorithm

The FPLS-DC algorithm includes a dimension reduction step and a nonlinear dependence step, aiming to characterize the dependence between the functional response variable Y(s) and a scalar predictor variable *X*. The FPLS-DC algorithm proceeds as follows:

**Step 1:** Estimate ${\hat{\psi }}_{1}(s),\ldots ,{\hat{\psi }}_{q}(s)$ using the sample (X,Y(s)) and the Equation (2) from Section 2.1. This step results in a low-dimensional informative projection vector (IPV) $Z={\left(Z_{1},\ldots ,Z_{q}\right)}^{T}$ of projections of Y(s) onto the basis functions ${\left\{{\hat{\psi }}_{j}(s)\right\}}_{j=1}^{q}$, where $Z_{j}=\int_{S}Y(s){\hat{\psi }}_{j}(s)ds$ for j=1,…,q.

**Step 2:** Optimize the empirical unbiased distance covariance:

(4) $$\underset{\gamma }{\max}\;{\text{dcov}}_{n}^{2}(X,Z^{T}\gamma )\;\;\;\;s.t.\;\;\;\;||\gamma |{|}_{2}=1.$$

The optimization problem (Equation 4) can be transformed into an unconstrained optimization problem by using a spherical coordinate transformation. This is because the constraint $\gamma^{T}\gamma =1$ corresponds to a hypersphere in *q*-dimensional space, which can be parameterized by spherical coordinates. In spherical coordinates, the parameter γ is represented by a vector θ of length q−1, where each $\theta_{i}$ signifies the angle between γ and the *i*th coordinate axis. The function J(θ) then transforms θ to γ, mapping the spherical coordinates to a point on the unit hypersphere.

Utilizing spherical coordinates, the optimization problem (Equation 4) can be reformulated as

(5) $$\underset{\theta \in R^{q-1}}{\max}{\text{dcov}}_{n}^{2}(X,Z^{T}J(\theta )).$$

This becomes an unconstrained nonconvex optimization problem that can be solved using standard optimization methods, such as simulated annealing. Simulated annealing is a stochastic optimization algorithm that finds the global maximum of a function by randomly adjusting the current solution and accepting moves that enhance the objective function with a probability that is governed by the temperature parameter.

Algorithm 1 presents a new feature screening procedure for all *p* components of X that is based on the optimization problem (Equation 5). This marginal screening approach assumes that the predictors are independent of each other. However, if there are correlations between predictors, then the FPLS-DC method may identify redundant variables instead of the most relevant ones. In the next section, we will present a novel iterative algorithm to address such redundancy.

Algorithm 1. FPLS-DC algorithm

**Input:** $(X_{1},\ldots ,X_{p},Y(s))={\left\{(X_{i1},\ldots ,X_{ip},Y_{i}(s))\right\}}_{i=1,\ldots ,n}$ and a positive integer *p*.

**Output:** The rank of importance score of $X_{1},\ldots ,X_{p}$.

1: $\hat{K}(s,t)=n^{-1}\sum_{i=1}^{n}[Y_{i}(s)-\bar{Y}(s)][Y_{i}(t)-\bar{Y}(t)]$, where $\bar{Y}(s)=n^{-1}\sum_{i=1}^{n}Y_{i}(s)$

2: **for**l=1,…,p**do**

3:  ${\hat{\psi }}_{1}^{\left(l\right)}(s)=n^{-1}\sum_{i=1}^{n}[Y_{i}(s)-\bar{Y}(s)](X_{il}-{\bar{X}}_{l})$, where ${\bar{X}}_{l}=n^{-1}\sum_{i=1}^{n}X_{il}$;

4:  **for**j=2,…,q**do**

5:   ${\hat{\psi }}_{j}^{\left(l\right)}(s)=\int_{S}{\hat{\psi }}_{j-1}^{\left(l\right)}(t)\hat{K}(s,t)dt;$

6:  Get the sequence $Z^{\left(l\right)}=(Z_{1}^{\left(l\right)},\ldots ,Z_{q}^{\left(l\right)})$ of projected Y(s) along ${\left\{{\hat{\psi }}_{j}^{\left(l\right)}(s)\right\}}_{j=1,\ldots ,q}$

7:  Compute the importance score of $X_{l}$ by optimizing

$$\underset{\theta \in R^{p-1}}{\max}{\text{dcov}}_{n}^{2}(X_{l},Z^{\left(l\right)}J(\theta ))$$

8: **return** Get the rank of importance score of $X_{1},\ldots ,X_{p}$.

### 3.2 I-FPLS-DC algorithm

We propose an iterative FPLS-DC algorithm, known as I-FPLS-DC, by introducing another constraint on X. Let γ and β be the coefficients of the response variables and predictor variables, respectively. For each β, we apply APLS algorithm to $\left(X^{T}\beta ,Y(s)\right)$ in order to compute the basis functions ${\hat{\psi }}_{1}(s),\ldots ,{\hat{\psi }}_{q}(s)$ and subsequently calculate the IPV vector $Z=(Z_{1},\ldots ,Z_{q})$ as a sequence of Y(s) along ${\left\{{\hat{\psi }}_{j}(s)\right\}}_{j\leq q}$. It is important to note that the IPV vector Z is a function of β.

For I-FPLS-DC, our optimization problem becomes:

(6) $$\underset{\gamma \in R^{q},\beta \in R^{p}}{\max}{\text{dcov}}_{n}^{2}(Z^{T}\gamma ,X^{T}\beta )\;\;s.t.\;\;\;||\gamma |{|}_{2}=1,||\beta |{|}_{2}=1.$$

Moreover, we can incorporate sparsity into (6) by adding an $L_{0}$ penalty term on the predictor variables, leading to:

(7) $$\underset{\gamma \in R^{q},\beta \in R^{p}}{\max}{\text{dcov}}_{n}^{2}(Z^{T}\gamma ,X^{T}\beta )\;s.t.\;\;\;||\gamma |{|}_{2}=||\beta |{|}_{2}=1,||\beta |{|}_{0}=K.$$

By using spherical coordinates β=J(α) and γ=J(θ), and selecting sparse variables in X, we can rewrite (7) as:

(8) $$\underset{\theta \in R^{q-1},\alpha \in R^{K-1}}{\max}{\text{dcov}}_{n}^{2}(Z^{T}J(\theta ),X_{A}^{T}J(\alpha )),$$

where A is the active set of X, consisting of selected features of size *K*. Optimizing Equation (8) can be computationally challenging when the total number of basis functions and significant variables are large. One strategy to manage this challenge involves fixing either the θ or α parameters and then updating the other set of parameters in an iterative manner.

Determining the *K* significant variables from *p* predictors remains a complex task. One intuitive approach is to rank the ${\text{dcov}}_{n}^{2}(X_{l},Y(s))$ for l=1,…,p and select the top-*K* variables. However, this method could be influenced by the correlation among predictor variables. An enhanced approach would be to update the importance score of each variable iteratively and constantly adjust the ranking of the significant variables to supersede the irrelevant ones. This method may provide a more accurate solution by considering the correlation among predictor variables. Given an estimated active set A, we can utilize two sacrifices to evaluate the importance of a variable:

$$\Delta_{l}=\{\begin{array}{c} {\text{dcov}}_{n}^{2}(Z^{T}\hat{\gamma },X_{A}^{T}\hat{\beta })-{\text{dcov}}_{n}^{2}(Z^{T}\hat{\gamma },X_{A\backslash l}^{T}\hat{\beta }),\;\;l\in A,\\ {\text{dcov}}_{n}^{2}(Z^{T}\hat{\gamma },X_{A\cup \{l\}}^{T}\hat{\beta })-{\text{dcov}}_{n}^{2}(Z^{T}\hat{\gamma },X_{A}^{T}\hat{\beta }),\;\;l\notin A, \end{array}$$

where $\hat{\gamma }$ and $\hat{\beta }$ are the solutions of Equation (7) based on the active set A.

The I-FPLS-DC procedure can be divided into two steps:

**Step 1:** Initialize an active set ${\hat{A}}_{\left(0\right)}$ by selecting the top-*K* variables based on ${\text{dcov}}_{n}^{2}(X_{l},Y(s))$.

**Step 2:** For each iteration j>0, update the active set ${\hat{A}}_{\left(j\right)}$ by selecting the top-*K* variables based on the updated magnitude $\Delta_{l}^{\left(j-1\right)}$ of each variable. Here, $\Delta_{l}^{\left(j-1\right)}$ is defined as:

(9) $$\begin{array}{c} \Delta_{l}^{\left(j-1\right)}=\{\begin{array}{c} {\text{dcov}}_{n}^{2}(Z^{T}\hat{\gamma },X_{{\hat{A}}_{\left(j-1\right)}}^{T}\hat{\beta })\\ -{\text{dcov}}_{n}^{2}(Z^{T}\hat{\gamma },X_{{\hat{A}}_{\left(j-1\right)}\backslash l}^{T}\hat{\beta })\;\;l\in {\hat{A}}_{\left(j-1\right)},\\ {\text{dcov}}_{n}^{2}(Z^{T}\hat{\gamma },X_{{\hat{A}}_{\left(j-1\right)}\cup \{l\}}^{T}\hat{\beta })\\ -{\text{dcov}}_{n}^{2}(Z^{T}\hat{\gamma },X_{{\hat{A}}_{\left(j-1\right)}}^{T}\hat{\beta })\;\;l\notin {\hat{A}}_{\left(j-1\right)}, \end{array} \end{array}$$

The detailed steps of the proposed I-FPLS-DC method are summarized in Algorithm 2. To enhance comprehension of Algorithms 1 and 2, we present the corresponding flowchart in Supplementary Fig. S1.

Algorithm 2. I-FPLS-DC algorithm

**Input:** $(X_{1},\ldots ,X_{p},Y(s))={\left\{(X_{i1},\ldots ,X_{ip},Y_{i}(s))\right\}}_{i=1,\ldots ,n}$ , the cardinality of active set *K*, the number of basis function *q*, and a positive integer $j_{\max}$.

**Output:** Active set $\hat{A^{*}}$.

1: Initialize the active set ${\hat{A}}_{\left(0\right)}$ based on top-K of ${\text{dcov}}_{n}^{2}(X_{l},Y(s))$, l=1,…,p.

2: **for**$j=1,\ldots ,j_{\max}$**do**

3:  Get the magnitude $\Delta_{l}^{\left(j-1\right)}$ of *l*th variable based on ${\hat{A}}_{\left(j-1\right)}$ through (9), l=1,…,p.

4:  Update the *j*th active set

$$\begin{array}{c} {\hat{A}}_{\left(j\right)}=\{l:\sum_{k=1}^{p}I(\Delta_{k}^{\left(j-1\right)}\leq \Delta_{l}^{\left(j-1\right)})\leq K\}. \end{array}$$

5:  **if**${\hat{A}}_{\left(j-1\right)}={\hat{A}}_{\left(j\right)}$**then** stop.

6: **Return**$\hat{A^{*}}={\hat{A}}^{\left(j\right)}$.

To determine the size of the active set in practice, information criteria such as Akaike information criterion (AIC) can be used. AIC provides a criterion for balancing the complexity of model estimation and the goodness of fit. However, when using $L_{2}$ penalty to constrain the maximization of unbiased distance variance, there may be an imbalance between the complexity of model estimation and the goodness of fit. To better determine the size of the active set, we integrate the sample size with the maximum distance covariance. For any active set A, we define a modified AIC as follows:

$$\begin{array}{c} \text{MAIC}=2|A|-n\text{ln}({\text{dcov}}_{\text{max}}), \end{array}$$

where ${\text{dcov}}_{\text{max}}=\max_{\theta \in R^{p-1},\alpha \in R^{|A|-1}}{\text{dcov}}_{n}^{2}(Z^{T}J(\theta ),X_{A}^{T}J(\alpha ))$.

### 3.3 Hypothesis testing

To explore the relationship between a functional response variable and a predictor, we conduct a hypothesis test for independence. Specifically, for l=1,…,q, we can write the null and alternative hypotheses as

(10) H0:Xl⊥⊥Y(s)   vs   H1:Xl⊥⊥Y(s),

To associate the predictor variables $X_{l}$ with the most pertinent regions of functional data Y(s), we can assign weights to each voxel *s* in the image Y(s) based on the directional influence of b(s). The signal pattern across S, which is linked to the variable $X_{l}$, can be elegantly captured by $\text{dcov}(X_{l},\int_{S}Y(s)b(s)ds)$. Consequently, the hypothesis test in (10) transforms into examining the independence between $X_{l}$ and $\int_{S}Y(s)b(s)ds$. Conventionally, a permutation procedure is used, but its computational demands can be prohibitive in large-scale GWAS. An alternative, as explored in prior literature (Gretton *et al.* 2005, Székely *et al.* 2007), entails approximating the null distribution of distance covariance through a gamma distribution. The shape and scale parameters of this distribution are estimated from ${\bar{\text{dcov}}}_{n}$, providing a practical and efficient approximation. The FPLS-DC test procedure can be divided into four parts:

**Step 1:** Calculate the empirical estimator ${\bar{\text{dcov}}}_{n}(X_{l},\int_{S}Y(s)\hat{b}(s)ds)$.

**Step 2:** Approximate ${\hat{b}}^{*}(s)$ using the FPLS-DC algorithm and compute the estimator ${\bar{\text{dcov}}}_{n}(X_{l},\int_{S}Y^{*}(s){\hat{b}}^{*}(s)ds)$, utilizing the permuted sample ($X_{l},Y^{*}(s)$), where $Y^{*}(s)=\{(Y_{i_{1}}((s)),\ldots ,Y_{i_{n}}(s))$ and $\left\{i_{1},\ldots ,i_{n}\right\}$ is the rearrangement of {1,…,n}.

**Step 3:** Repeat Step 2 for *M* times, resulting in *M* estimates ${\left\{{\bar{\text{dcov}}}_{n}(X_{l},\int_{S}Y^{*}(s){\hat{b}}_{\left(m\right)}^{*}(s)ds)\right\}}_{m=1,\ldots ,M}$, and subsequently calculate the scalar and shape parameters of the gamma distribution.

**Step 4:** Calculate the *P*-value, denoted as $\hat{p}$, and reject $H_{0}$ if $\hat{p}<\alpha$, where α is the prespecified significant level.

During GWAS with the FPLS-DC test, we can simplify the approximation of the gamma distribution parameters to expedite the testing speed. For a reference variable ($\tilde{X},Y(s)$), the gamma distribution’s mean and variance hinge solely on $\frac{1}{n^{4}}\sum_{i,k=1}^{n}|{\tilde{X}}_{i}-{\tilde{X}}_{k}|$ and ${\bar{\text{dcov}}}_{n}^{2}(\tilde{X},\tilde{X})$. For the testing variable ($X_{l},Y(s)$), adjust by substituting $\sum_{i,k=1}^{n}|{\tilde{X}}_{i}-{\tilde{X}}_{k}|$ with $\sum_{i,k=1}^{n}|X_{il}-X_{kl}|$ and ${\bar{\text{dcov}}}_{n}^{2}(\tilde{X},\tilde{X})$ with ${\bar{\text{dcov}}}_{n}^{2}(X_{l},X_{l})$. Applying this, we can estimate gamma distribution mean and variance of ($X_{l},Y(s)$). Then, we can estimate the null distribution of ($X_{l},Y(s)$).

Similar to the FPLS-DC test, I-FPLS-DC can conduct group genetics testing following (7). The details are summarized in the supplemental material. In the context of the I-FPLS-DC test procedure, which focuses on a conditional test given an active set A, we define our hypotheses as follows:

(11) H0:Xl⊥⊥Y(s)|XA\{l}   vs   H1:Xl⊥⊥Y(s)|XA\{l}.

Then, the I-FPLS-DC test procedure exhibits slight variations depending on the specific variable. Assuming that we have obtained the estimated direction $\hat{b}(s)$ and the estimated active set $\hat{A}$ through the I-FPLS-DC method, the following steps are followed: For the variable *X*, the procedure closely resembles the FPLS-DC process, with a minor modification in Step 3. If $l\in \hat{A}$, the adjustment lies in the determination of ${\hat{b}}_{\left(m\right)}^{*}(s)$ during Step 3. We optimize the objective function (8) using the permuted sample ($X,Y^{*}(s)$) and the estimated active set $\hat{A}$ to compute ${\hat{b}}_{\left(m\right)}^{*}(s)$. Conversely, if $l\notin \hat{A}$, the only variation is in setting the value of ${\hat{b}}_{\left(m\right)}^{*}(s)$ during Step 3, where it is set to be $\hat{b}(s)$.

## 4 Simulation

In this section, we evaluate the finite sample performance of FPLS-DC and I-FPLS-DC through several numerical experiments. We set four types of examples to contrast the performances of these methods against APLS (Delaigle and Hall 2012), wdCor (Wen *et al.* 2020), and FGWAS (Huang *et al.* 2017). The comparison is conducted using two evaluation metrics:

- The quantile of the minimum number of chosen variables that encompasses all the causal variables.
- The sensitivity rate, defined as the proportion of significant causal SNPs over the total number of causal SNPs, and the nonsensitivity rate, defined as the ratio of significant noncausal SNPs to the total number of noncausal SNPs, with significance levels of 0.001, 0.01, and 0.05.

In our comparisons, we apply sample sizes of 200, while the dimension of genetic data is set at 1000 or 2000 across all scenarios. We perform 100 Monte Carlo simulations in each case.

Example 1: We generate Y(s) as follows:

$$\begin{array}{c} Y(s)=\beta_{0}(s)+X^{*T}\beta^{*}(s)+\eta (s)+e(s),\;s\in [0,1], \end{array}$$

where $\beta_{0}(s)=0.1\;\text{exp}(-{\left(s-0.5\right)}^{2})I(s\in [0,1])$ and $X^{*}={\left(X_{1},X_{2},X_{6},X_{11}\right)}^{T}$. Moreover, $\beta^{*}(s)={\left(\beta_{1}(s),\beta_{2}(s),\beta_{6}(s),\beta_{11}(s)\right)}^{T}$, where $\beta_{k}(s)=c_{k}(\text{exp}(-{\left(s-0.5\right)}^{2}/2)-0.98)I(s\in [0.25,0.50])$ for k=1,2,6, and 11, in which $c_{1}=-120$, $c_{2}=100$, $c_{6}=-90$, and $c_{11}=90$. The function η(s) denotes a random effect function that characterizes subject-specific spatial variation, while e(s) symbolizes measurement error. Let $GP(\mu (s),\Sigma (s,s^{\prime }))$ denote a Gaussian process with mean function μ(s) and covariance function $\Sigma (s,s^{\prime })$. The e(s) is generated from $GP(0,0.5I(s=s^{\prime }))$ and η(s) is generated from $GP(0,0.5^{|s-s^{\prime }|+1})$. We put the number of grid points 200 evenly in [0,1].

The genetic data $X={\left(X_{1},\ldots ,X_{p}\right)}^{T}$, where p=1000 or 2000, is simulated as follows: Initially, *n* subjects are created by randomly combining haplotypes of HapMap CEU subjects. We then use PLINK software to establish linkage disequilibrium (LD) blocks, based on the genotypes of these simulated subjects. We randomly select 200 or 400 blocks from the resultant LD blocks and amalgamate the haplotypes of HapMap CEU subjects within each chosen block to create genotype variables for these subjects. Finally, 5 SNPs are randomly selected from each block to formulate the genetic data *X*.

Example 2: We use the same setting in Example 1 except that Y(s) is generated as follows:

$$\begin{array}{c} Y(s)=\beta_{0}(s)\;+\;{\left(X^{*T}\beta^{*}(s)\right)}^{2}\;+\;\eta (s)\;+\;e(s),\;s\in [0,1]. \end{array}$$

Example 3: We generate Y(s) for $s=(s_{x},s_{y})\in [0,20]\times [1,10]$ as follows:

$$\begin{array}{c} Y(s)={\left[X^{*T}\beta^{*}(s)\right]}^{2}h(s)+e(s), \end{array}$$

where $X^{*}={\left(X_{1},X_{2},X_{3},X_{30}\right)}^{T}$ and $h(s)=I\{{\left(s_{x}-10\right)}^{2}+{\left(s_{y}-5\right)}^{2}\leq 5\}$. Moreover, $\beta^{*}(s)={\left(\beta_{1}(s),\beta_{2}(s),\beta_{3}(s),\beta_{30}(s)\right)}^{T},$ where

$$\begin{array}{c} \beta_{k}(s)=0.8\sqrt{{\left(s_{x}-10\right)}^{2}+{\left(s_{y}-5\right)}^{2}}\;\;\;\text{for}\;\;\;k=1,2,3 \end{array}$$

and $\beta_{30}=0.24\sqrt{{\left(s_{x}-10\right)}^{2}+{\left(s_{y}-5\right)}^{2}}$. The e(s) is simulated as $e(s)=\xi_{1}\phi_{1}(s)+\xi_{2}\phi_{2}(s)$, where $\phi_{1}(s)=\sqrt{0.5}\;\text{sin}(2\pi s_{x}/25),\;\phi_{2}(s)=\sqrt{0.5}\;\text{cos}(2\pi s_{y}/20)$, $\xi_{1}=\lambda_{1}Z_{1},\;\xi_{2}=\lambda_{2}Z_{2},\;\lambda_{1}=\sqrt{1.2},\;\lambda_{2}=\sqrt{1.0}$, and $Z_{1}$ and $Z_{2}$ are independent standard normal random variables.

The genetic data $X=(X_{1},\ldots ,X_{p})$ with p=1000 or 2000 are generated as follows. Firstly, we generate multivariate normal data Z∼N(0,∑), where 0 is a q×1 vector of zeros and Σ is a covariance matrix with elements $\sum_{ij}=0.5^{\left|i-j\right|}$. Secondly, we transform $Z_{i}$ into the genetic data $X_{i}=I(-0.44\leq Z_{i}\leq 0.44)+2\times I(Z_{i}\geq 0.44)$, indicating $P(X_{i}=0)=P(Z_{i}\leq -0.44)$, $P(X_{i}=1)=P(-0.44\leq Z_{i}\leq 0.44)$, and $P(X_{i}=2)=P(Z_{i}\geq 0.44)$.

Example 4: We generate Y(s) as follows:

$$\begin{array}{c} Y(s)={\left[X^{*T}\beta^{*}(s)\right]}^{2}+\eta (s)+e(s),\;s\in [0,1], \end{array}$$

where $\beta^{*}(s)={\left(\beta_{1}(s),\beta_{2}(s),\beta_{3}(s),\beta_{30}(s)\right)}^{T}$, in which $\beta_{k}(s)=2/[\sqrt{13}s]I(s\in [0.25,0.75])$ for k=1,2, and 3 and $\beta_{30}(s)=1/[\sqrt{13}s]I(s\in [0.25,0.75]).$ Moreover, $X^{*}={\left(X_{1},X_{2},X_{3},X_{30}\right)}^{T}$. The genetic data $X=(X_{1},\ldots ,X_{p})$ with p=1000 or 2000 are generated from multivariate normal distributions N(0,Σ), where the covariance matrix Σ has elements $\sigma_{ij}=0.5^{\left|i-j\right|}$ for i,j=1,…,p. The noise e(s) is generated from a Gaussian process $GP(0,I(s=s^{\prime }))$, and the function η(s) is generated from a Gaussian process $GP(0,0.5^{\left|s-s^{\prime }\right|})$. Notably, both are independent of the genetic data.

Tables 1 presents intriguing insights into the performance of various methods in the four examples. The results derived from the minimum model size reveal that I-FPLS-DC surpasses all other methods in discerning the most consequential variables while excluding irrelevant ones. FPLS-DC also displays its effectiveness by maintaining a smaller model size compared to the wdCor method in the majority of the examples. The wdCor method falls short of the performance levels exhibited by both FPLS-DC and I-FPLS-DC across both sample sizes. In contrast, APLS and FVGWAS tend to incorporate more variables into the selected models, particularly when dealing with smaller sample sizes.

**Table 1.** The 5%, 25%, 50%, 75%, and 90% quantiles of the minimum model size for Examples 1–4.

*p*		1000					2000				
Example	Method	5%	25%	50%	75%	90%	5%	25%	50%	75%	90%
1	FPLS-DC	5.95	9.00	11.00	29.25	302.00	5.00	13.00	62.50	136.00	210.00
	I-FPLS-DC	4.00	6.00	9.00	18.00	202.00	4.00	7.00	9.50	85.00	194.60
	wdCor	5.95	9.00	15.00	98.00	343.90	6.00	20.75	152.00	342.25	668.50
	APLS	70.65	287.00	478.00	766.50	858.30	135.00	716.75	1238.50	1656.00	1889.00
	FVGWAS	6.50	191.00	535.50	748.00	908.00	10.00	509.50	1512.00	1994.5	1999.50
2	FPLS-DC	9.00	27.00	67.50	255.25	465.60	7.45	32.50	108.00	225.00	410.20
	I-FPLS-DC	4.00	7.00	22.50	193.25	263.10	7.00	19.25	68.50	211.50	403.30
	wdCor	11.95	36.75	84.50	226.25	396.20	10.00	32.25	140.50	355.00	627.80
	APLS	207.90	601.25	753.00	899.75	972.20	610.35	1215.25	1489.00	1811.75	1925.20
	FVGWAS	62.50	456.50	729.00	859.00	911.50	679.50	1141.50	1670.50	1909.50	1991.50
3	FPLS-DC	5.00	15.75	45.50	93.25	142.20	5.00	15.25	64.50	135.25	264.00
	I-FPLS-DC	4.00	5.00	8.00	30.00	124.80	4.00	5.00	21.00	124.25	263.40
	wdCor	6.00	43.00	102.50	248.00	399.30	6.95	37.50	104.50	505.25	866.30
	APLS	57.10	270.50	504.00	774.25	957.30	54.65	501.75	894.50	1509.50	1883.30
	FVGWAS	148.50	318.50	548.00	720.50	854.50	1178.00	1691.00	1886.50	1962.50	1990.50
4	FPLS-DC	33.00	260.50	433.00	680.75	890.20	86.85	302.75	792.00	1324.50	1572.10
	I-FPLS-DC	4.00	4.00	4.00	4.00	10.80	4.00	4.00	4.00	22.00	366.40
	wdCor	32.00	210.25	454.00	772.75	863.10	78.95	263.25	703.00	1223.75	1558.80
	APLS	500.45	654.75	797.00	891.00	964.20	883.70	1291.00	1576.50	1842.75	1922.30
	FVGWAS	49.00	186.50	395.00	727.50	848.00	506.00	947.50	1392.50	1775.50	1914.50

Table 2 presents the sensitivity rates of all methods. It suggests that FPLS-DC and I-FPLS-DC are highly likely to accurately identify important variables, with wdCor demonstrating comparable performance, albeit slightly inferior to FPLS-DC and I-FPLS-DC. Both APLS and FVGWAS tend to manifest lower sensitivity rates, implying they might incorporate fewer pertinent variables in their selected models. However, all methods exhibit similar performance when it comes to irrelevant variables, which indicates the stability of their tests when dealing with such variables. The decision to use a gamma distribution as an approximation in our test is due to the complexities of the null distribution, potentially resulting in higher NSR of FPLS-DC and I-FPLS-DC at times.

**Table 2.** Significant rates (SR) and nonsignificant rates (NSR) for Examples 1–4.

*p*		1000						2000					
Example	Method	0.001		0.01		0.05		0.001		0.01		0.05	
		SR	NSR	SR	NSR	SR	NSR	SR	NSR	SR	NSR	SR	NSR
1	FPLS-DC	0.905	0.018	0.945	0.035	0.975	0.068	0.937	0.010	0.965	0.027	0.978	0.061
	I-FPLS-DC	0.914	0.012	0.965	0.028	0.975	0.068	0.915	0.009	0.960	0.024	0.985	0.063
	wdCor	0.763	0.006	0.833	0.016	0.908	0.058	0.498	0.005	0.550	0.011	0.613	0.038
	APLS	0.213	0.002	0.335	0.010	0.455	0.053	0.290	0.002	0.438	0.014	0.570	0.068
	FVGWAS	0.292	0.001	0.503	0.003	0.640	0.048	0.228	0.001	0.310	0.002	0.430	0.072
2	FPLS-DC	0.567	0.005	0.763	0.018	0.884	0.069	0.533	0.002	0.723	0.011	0.867	0.061
	I-FPLS-DC	0.646	0.009	0.783	0.025	0.886	0.064	0.653	0.003	0.785	0.023	0.868	0.063
	wdCor	0.455	0.004	0.640	0.013	0.803	0.054	0.308	0.002	0.545	0.013	0.660	0.057
	APLS	0.028	0.001	0.073	0.010	0.163	0.050	0.023	0.001	0.053	0.007	0.1323	0.039
	FVGWAS	0.310	0.001	0.395	0.004	0.468	0.027	0.138	0.001	0.185	0.003	0.275	0.037
3	FPLS-DC	0.770	0.003	0.820	0.018	0.925	0.079	0.785	0.002	0.835	0.013	0.918	0.059
	I-FPLS-DC	0.813	0.007	0.863	0.023	0.925	0.063	0.808	0.006	0.860	0.021	0.922	0.061
	wdCor	0.768	0.002	0.803	0.020	0.828	0.059	0.770	0.002	0.808	0.011	0.873	0.048
	APLS	0.678	0.006	0.720	0.026	0.765	0.094	0.625	0.002	0.715	0.014	0.778	0.068
	FVGWAS	0.010	0.001	0.063	0.012	0.243	0.052	0.003	0.001	0.010	0.010	0.058	0.050
4	FPLS-DC	0.565	0.000	0.720	0.006	0.770	0.072	0.642	0.001	0.740	0.010	0.773	0.067
	I-FPLS-DC	0.614	0.009	0.748	0.026	0.778	0.063	0.750	0.006	0.805	0.023	0.842	0.057
	wdCor	0.667	0.001	0.748	0.010	0.770	0.046	0.702	0.001	0.740	0.011	0.770	0.054
	APLS	0.000	0.002	0.020	0.023	0.060	0.075	0.000	0.001	0.013	0.011	0.063	0.050
	FVGWAS	0.373	0.000	0.585	0.006	0.693	0.041	0.438	0.000	0.628	0.007	0.738	0.044

In Supplementary Fig. S2, a detailed comparison of computation times for various methods across 100 repeated simulations under the null hypothesis is shown. The analysis reveals that FVGWAS is the most time-efficient method. However, FPLS-DC and I-FPLS-DC also demonstrate commendable speed, significantly surpassing wdCor and APLS in computational efficiency. This evidence implies that integrating FPLS-DC and I-FPLS-DC into existing analysis workflows could enhance the speed of detecting genetic variants linked to complex diseases in extensive genetic association studies.

## 5 UK Biobank data analysis

In this section, we apply our FPLS-DC framework to analyze hippocampus surface data obtained from phases 1 and 2 of the UK Biobank (UKB). The UKB’s principal goal is to collect extensive biomedical information from around 500 000 participants. This initiative aims to assess the influence of genetics, lifestyle choices, and environmental factors on various diseases and health conditions. Our analysis uses a dataset comprising 10 000 unrelated individuals of British ancestry, encompassing two key components. The first component includes the left and right hippocampus radial distances for each subject, with each surface represented by 15 000 vertices. This detailed representation allows for a comprehensive analysis of the hippocampal structure. The second component consists of SNPs located on 22 autosomes. The primary objective of this data analysis is to explore the genetic impact of these SNPs on the hippocampus. Given the hippocampus’s pivotal role in memory processes, our study focuses on understanding how genetic variations might influence hippocampal structure and function, and consequently, memory lapses. This research is instrumental in deepening our understanding of the genetic underpinnings of memory-related aspects of the brain.

We processed the MRI data using standard procedures, including AC-PC correction, skull-stripping, cerebellum removal, intensity inhomogeneity correction, segmentation, and registration. This was followed by automatic regional labeling of the template, transferred to subject images through deformable registration, enabling the computation of volumes for 93 ROIs per subject. For the hippocampus, we used a subregional analysis package utilizing surface fluid registration (Shi *et al.* 2013), which leverages isothermal coordinates and fluid registration for hippocampal surface mapping. This allowed us to calculate various surface statistics, such as multivariate tensor-based morphometry and radial distance measures on the registered surfaces. Further methodological details are available in Wang *et al.* (2011).

We downloaded the UKB imputed genotype data and applied the following standard quality control procedures: excluding subjects with more than 10% missing genotypes, only including SNPs with MAF>0.01, genotyping rate >90%, and passing Hardy-Weinberg test (*P*-value > $1\times 10^{-7}$). After quality control, we limit our analysis to 653, 122 SNPs that overlap with the HapMap3 reference panel (International HapMap 3 Consortium et al. 2010) to balance accuracy and computational burden (Ge *et al.* 2019).

We adjusted for key covariates in our study, including the top 20 genetic PCs, age at imaging, gender, interaction terms (age × gender, age^2^, age^2^ × gender), education, study site, motion, image quality, scale, and brain position. To minimize their impact on the FPLS-DC analysis, we preprocessed the imaging data by consolidating IDs into one file and imputing missing data with subject-specific mean values. The residualized imaging data was then calculated using the formula the equation $[I-X{\left(X^{T}X\right)}^{-1}X^{T}]Y$, where *X* is the design matrix (enhanced by adding a constant column before the covariate matrix) and *Y* is the image data. Given the longer processing time for wdCor and APLS tests on real data, the residualized data, along with the genetic data, were analyzed using FPLS-DC, I-FPLS-DC, and FVGWAS methods.

Supplementary Table S1 provides an overview of the top 10 SNPs that have been detected by FPLS-DC, I-FPLS-DC, and FVGWAS in both the right and left hippocampus. These results were obtained with a significance level at $7.655\times 10^{-8}$, which corresponds to the threshold of 0.05 divided by the total number of SNPs (653 171). Within the findings generated by FPLS-DC, as highlighted in Table 1, SNP rs11245347 exhibited the highest significance with a *P*-value of $9.992\times 10^{-15}$. This SNP, located on the *FAM53B* gene, demonstrates a remarkable association with the right hippocampus. In addition, SNP rs9321028 on the *TPD52L1* gene exhibited a significant association with the left hippocampus (*P*-value $=9.881\times 10^{-15}$). Liu *et al.* (2023) indicated that SNP rs11245347 (chr10) showed significant effects on several hippocampal and subfield volumes in both EAS and EUR, including the right hippocampal tail volume. Zhao *et al.* (2019) and Van der Meer *et al.* (2020) both found significant gene-level associations between *FAM53B* and hippocampal subfield volumes.

In the I-FPLS-DC results detailed in Supplementary Table S1, two prominent SNPs stand out: rs853169 and rs7998301, each displaying a remarkably low *P*-value of 1.110$\times 10^{-16}$. These SNPs, respectively situated in the *ARHGAP26* and *SPATA13* genes, showcase the most robust relationships with the right and left hippocampus among the identified SNPs. Specifically, the *ARHGAP26* gene, associated with SNP rs853169, has been observed to exhibit expression in a distinct subset of hippocampal neurons, as highlighted by Jarius *et al.* (2015) and Uhlén *et al.* (2015).

Unlike FVGWAS, which fails to identify any SNPs with *P*-values below $7.655\times 10^{-8}$, FPLS-DC has discovered 142 significant SNPs linked to the right hippocampus and 257 to the left hippocampus. I-FPLS-DC, on the other hand, has identified 222 significant SNPs associated with the right hippocampus and 220 with the left hippocampus. Moreover, a Venn diagram in Supplementary Fig. S5 illustrates that FPLS-DC and I-FPLS-DC share 55 significant SNPs connected to the left hippocampus and 60 to the right hippocampus. The overlap of significant SNPs detected by both methods is detailed in Supplementary Tables S2 and S3 (left hippocampus) and Supplementary Tables S4 and S5 (right hippocampus). Within these overlaps, genes such as *FAM53B, ADAMTS18*, and *Tll-1*, among others, are implicated in hippocampal function and have been previously mentioned in literature such as Zhao *et al.* (2019), Van der Meer *et al.* (2020), Zhu *et al.* (2019), Tamura *et al.* (2005), and other pertinent studies. Remarkably, I-FPLS-DC is capable of identifying SNPs with *P*-values below $10^{-15}$, a sensitivity not shared by FPLS-DC or FVGWAS.

**Figure 1.**
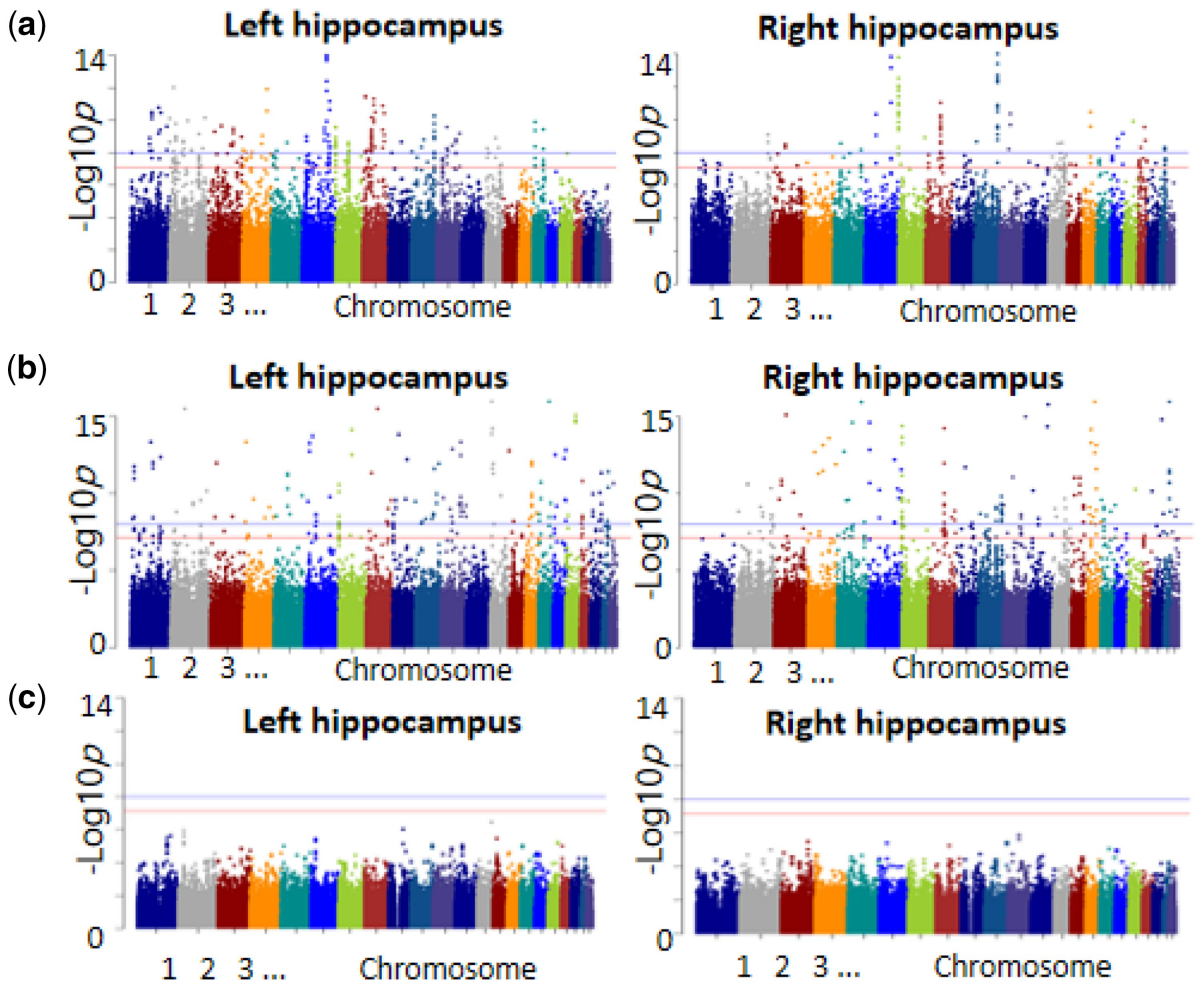
Comparative analysis of GWAS for left and right morphological features: (a) FPLS-DC, (b) I-FPLS-DC, and (c) FVGWAS

In addition, we provide Manhattan plots (Fig. 1) created using FPLS-DC, I-FPLS-DC, and FVGWAS to highlight significant SNPs. Supplementary Fig. S3a focuses on the influence of the most notable SNPs, rs9321028 and rs11245347, identified by FPLS-DC, on the left and right hippocampus. Supplementary Figure S3b depicts the effect of the most significant SNPs, rs7998301 and rs85316, discovered by I-FPLS-DC, on the left and right hippocampus. It is clear from Supplementary Fig. S3 that I-FPLS-DC pinpoints subregions linked to the most significant SNPs similarly to FPLS-DC. Supplementary Fig. S4 demonstrates that our proposed methods achieve notably lower *P*-values when testing significant SNPs compared to those yielded by FVGWAS. Furthermore, we examined the polygenic effects of each active set derived from individual chromosomes (Supplementary Table S6) and conducted a pathway enrichment analysis (Supplementary Fig. S6) utilizing the Gene Ontology analysis approach. From the result of Supplementary Fig. S6, it can be observed that both FPLSDC and I-FPLS-DC jointly identify genes associated with ‘axon extension involved in axon guidance,’ ‘juxtaparanode region of axon,’ and so on. Those pathways are both related to the hippocampus in human beings through their roles in neural circuit development and function.

## 6 Conclusion

Our research introduces two powerful techniques, FPLS-DC and I-FPLS-DC, for assessing genetic markers in high-dimensional imaging data. These methods use advanced statistical approaches to handle complex relationships between genetic and imaging data. FPLS-DC uses a thorough screening process and permutation testing, while I-FPLS-DC uses an iterative splicing technique for nonlinear model analysis. Our findings show these strategies efficiently handle ultra-high dimensional imaging genetic data, expanding the scope of nonlinear genetic analysis.

To enhance computational efficiency, future work could focus on developing faster approximations for estimating the null distributions of FPLS-DC and I-FPLS-DC, speeding up whole-genome analyses of whole-brain data. In addition, extending these methods to handle more complex models, such as graph, longitudinal, and Riemannian manifold models, would enable researchers to explore intricate interactions between genetic variants and imaging phenotypes. Furthermore, while currently applied to MRI, FPLS-DC and I-FPLS-DC have the potential to extend to other imaging data types like CT, PET, and EEG. This extension would broaden the range of data available, providing a more comprehensive understanding of the genetic basis of complex disorders, such as schizophrenia.

## Supplementary Material

btae173_Supplementary_Data
