# Supplementary material for: FPLS-DC: functional partial least squares through distance covariance for imaging genetics
Source: Bioinformatics. 2024 Mar 29;40(4):btae173. doi: 10.1093/bioinformatics/btae173 (PMC11034987; doi:10.1093/bioinformatics/btae173)
Supplement: btae173_Supplementary_Data [file btae173_supplementary_data.pdf]

Supplementary material of "FPLS-DC: Functional partial least squares through distance covariance for imaging genetics"

Wenliang Pan[&,1],Yue Shan[&,2],Chuang Li[&,3],Shuai Huang[2],Tengfei Li[4], Yun Li[2], and Hongtu Zhu[*,2]

1. Key Laboratory of Systems and Control, Academy of Mathematics and Systems Science, Chinese Academy of Sciences, Beijing 100190, China

2. Departments of Biostatistics, Statistics, Genetics, and Computer Science and Biomedical Research Imaging Center, University of North Carolina, Chapel Hill

3. School of Mathematics, Sun Yat-sen University, Guangzhou, China

4. Departments of Radiology and Biomedical Research Imaging Center, University of North Carolina, Chapel Hill

Many-to-many imaging-genetics test.

For the many-to-many imaging-genetics testing, the null and alternative hypothesis is

$$H_0 : X \perp\!\!\!\perp Y(s) \quad \text{vs} \quad H_1 : X \not\perp\!\!\!\perp Y(s). \tag{1}$$

The detail of test process is summarised as follows:

Step 1: Calculate the empirical estimator $\overline{\text{dcov}}_n(\mathbf{X}^T\beta, \int_S \mathbf{Y}(s)\widehat{b}(s)ds)$ through I-FPLS-DC and get the activate set $\widehat{\mathcal{A}}$.

Step 2: Calculate the estimator $\overline{\text{dcov}}_n(\mathbf{X}^T\beta^*, \int_S \mathbf{Y}^*(s)\widehat{b}^*(s)ds)$ through optimization (7) with $\|\beta\|_0 = |\widehat{\mathcal{A}}|$. Note that $\mathbf{Y}^*(s)$ is obtained through Step 2 of FPLS-DC test process.

Step 3: Repeat Step 2 for M times, resulting in M estimators $\{\overline{\text{dcov}}_n(\mathbf{X}^T\beta^*_{(m)}, \int_S \mathbf{Y}^*(s)\widehat{b}^*_{(m)}(s)ds)\}_{m=1,\dots,M}$, and get the scalar and shape parameters of the gamma distribution.

Step 4: Calculate the p-value, denoted as $\widehat{p}$, and reject H_0 if $\widehat{p} < \alpha$, where α is the pre-specified significant level.

Table S1: The top 10 SNPs identified by FPLS-DC, I-FPLS-DC and FVGWAS for the left and right hippocampus.

Method	Left hippocampus					Right hippocampus				
	SNP	gene	CHR	Alleles	p.value	SNP	gene	CHR	Alleles	p.value
FPLS-DC	rs9321028	TPD52L1	6	T/A/G	9.881×10^{-15}	rs11245347	FAM53B	10	G/A/C/T	9.992×10^{-15}
	rs6916559	TPD52L1	6	G/A	1.366×10^{-14}	rs4896079	None	6	A/G	1.421×10^{-14}
	rs2273560	TPD52L1	6	C/T	2.043×10^{-14}	rs2533878	GNA12	7	G/A/C/T	1.577×10^{-14}
	rs3799743	TPD52L1	6	G/A/C	3.657×10^{-13}	rs10901809	FAM53B	10	C/T	3.897×10^{-14}
	rs17044517	None	2	T/G	8.933×10^{-13}	rs4896077	None	6	G/T	6.672×10^{-14}
	rs17689892	TLL1	4	A/C/T	1.161×10^{-12}	rs7099316	EEF1AKMT2	10	G/A/C/T	1.908×10^{-13}
	rs565228	TPD52L1	6	G/A	1.478×10^{-12}	rs10901818	EEF1AKMT2	10	G/A/C/T	2.074×10^{-13}
	rs3823222	TPD52L1	6	G/A	1.567×10^{-12}	rs798490	GNA12	7	G/A/C	2.959×10^{-13}
	rs4289816	TNKS	8	T/C	3.088×10^{-12}	rs7898003	EEF1AKMT2	10	G/A	3.605×10^{-13}
	rs17734024	TNKS	8	G/A/T	3.766×10^{-12}	rs11245357	EEF1AKMT2	10	A/C/G	4.782×10^{-13}
I-FPLS-DC	rs7998301	SPATA13	13	G/T	1.110×10^{-16}	rs853169	ARHGAP26	5	G/A/C	1.110×10^{-16}
	rs1346563	ADAMTS18	16	G/A	1.111×10^{-16}	rs12427363	None	12	T/C	1.112×10^{-16}
	rs6738783	SNRNP27	2	A/C/G	3.331×10^{-16}	rs2278545	SMAD3	15	T/C/G	1.210×10^{-16}
	rs4598240	None	8	G/A	3.332×10^{-16}	rs16998444	IGSF5	21	G/A	1.710×10^{-16}
	rs3809971	ALPK2	18	C/G/T	8.882×10^{-16}	rs11919532	C3orf49	3	C/A/T	7.772×10^{-16}
	rs9962964	ALPK2	18	C/A/T	1.665×10^{-15}	rs7944144	None	11	C/A/T	9.992×10^{-15}
	rs3809970	ALPK2	18	C/T	2.220×10^{-15}	rs6021649	None	20	A/G/T	1.554×10^{-15}
	rs9962203	ALPK2	18	G/A/T	2.554×10^{-15}	rs10038508	None	5	G/A/C/T	2.442×10^{-15}
	rs17821569	POLR1D	13	C/G/T	6.328×10^{-15}	rs7768618	None	6	A/G/T	2.554×10^{-15}
	rs11509864	None	7	C/T	7.772×10^{-15}	rs1636249	GNA12	7	T/G	4.108×10^{-15}
FVGWAS	rs9514252	None	13	A/G	3.621×10^{-7}	rs11231727	FKBP2	11	C/G/T	1.356×10^{-6}
	rs11142017	SPATA31E1	9	G/A/C	9.742×10^{-7}	rs3741403	DNAJC4	11	C/G/T	2.351×10^{-6}
	rs935996	LTBP1	2	A/C/T	1.177×10^{-6}	rs4680035	None	3	A/C/G	3.434×10^{-6}
	rs7575377	LTBP1	2	G/T	2.193×10^{-6}	rs11245347	FAM53B	10	G/A/C/T	3.674×10^{-6}
	rs6668056	GPR25	1	C/T	2.397×10^{-6}	rs10901818	EEF1AKMT2	10	G/A/C/T	4.024×10^{-6}
	rs41464348	GPR25	2	C/T	2.431×10^{-6}	rs1055256	EEF1AKMT2	10	G/A/C/T	4.086×10^{-6}
	rs438148	None	1	C/A/G/T	3.261×10^{-6}	rs1214752	ABCC10	6	C/G/T	4.143×10^{-6}
	rs1761013	LINC00871	14	C/G/T	3.323×10^{-6}	rs7898003	EEF1AKMT2	10	G/A	4.227×10^{-6}
	rs4713099	ZNF184	6	T/G	3.709×10^{-6}	rs7099316	EEF1AKMT2	10	G/A/C/T	4.377×10^{-6}
	rs4713098	ZNF184	6	C/T	5.151×10^{-6}	rs11245357	EEF1AKMT2	10	A/C/G	4.794×10^{-6}

Table S2: The significant SNPs identified by FPLS-DC and I-FPLS-DC for the left hippocampus (a).

method				FPLS-DC	I-FPLS-DC
snp	gene	CHR	Alleles	p.value	
rs10023933	THEGL	4	G/A	1.74E-08	2.29E-10
rs10067993	None	5	A/G/T	6.39E-08	2.86E-09
rs10200577	LINC01934	2	T/C	7.14E-08	1.21E-08
rs10476508	None	5	T/A/C	2.33E-08	1.39E-10
rs10489676	None	1	T/C	7.23E-09	3.89E-09
rs10496175	MXD1	2	T/A/C/G	2.10E-09	6.44E-08
rs10496773	THSD7B	2	T/C	7.44E-09	4.09E-10
rs10496775	THSD7B	2	T/G	7.00E-09	5.54E-10
rs11102374	None	1	G/A/C	7.18E-09	4.64E-14
rs11245347	FAM53B	10	G/A/C/T	2.04E-08	1.14E-12
rs1182179	GNA12	7	A/G	6.13E-08	7.09E-09
rs1182188	GNA12	7	T/C	3.39E-08	8.30E-09
rs11825265	None	11	C/G/T	1.99E-09	6.60E-10
rs11933465	ADGRA3	4	A/C/G	2.96E-08	5.56E-08
rs12782006	LOC101929727	10	T/C	3.67E-08	4.79E-09
rs12804187	None	11	A/G	9.98E-09	1.33E-09
rs12804616	None	11	G/A	1.10E-08	6.97E-10
rs1346563	ADAMTS18	16	G/A	3.67E-10	1.11E-16
rs1636250	GNA12	7	T/C/G	4.30E-09	2.00E-08
rs17020606	None	3	G/A	5.12E-10	1.11E-12
rs17044517	None	2	T/G	8.93E-13	2.27E-08
rs17112466	None	11	A/C/G/T	2.24E-09	3.07E-09
rs17365411	MIR4437	2	T/C	2.65E-08	2.89E-10
rs17424113	None	8	C/A/G/T	1.97E-09	7.90E-09
rs17625338	None	4	T/C/G	8.87E-10	3.78E-09
rs17633422	TLL1	4	C/T	2.68E-11	5.98E-08
rs17671830	None	16	C/T	7.36E-08	4.51E-08
rs17689892	TLL1	4	A/C/T	1.16E-12	8.32E-10
rs17734024	TNKS	8	G/A/T	3.77E-12	2.81E-08
rs17821569	POLR1D	13	C/G/T	1.99E-08	6.33E-15
rs1943505	LOC105369439	11	C/G/T	3.64E-09	4.13E-08
rs1969742	None	1	C/G/T	3.07E-11	1.03E-12
rs1978628	ADAMTS18	16	A/C/G	5.80E-09	1.30E-08
rs2273560	TPD52L1	6	C/T	2.04E-14	1.76E-10
rs2533878	GNA12	7	G/A/C/T	3.00E-08	7.54E-11
rs2758688	None	1	C/A/G/T	4.16E-11	1.12E-12
rs2777987	None	1	A/C/G/T	7.83E-11	2.14E-12

Table S3: The significant SNPs identified by FPLS-DC and I-FPLS-DC for the left hippocampus(b).

method				FPLS-DC	I-FPLS-DC
snp	gene	CHR	Alleles	p.value	
rs4332745	None	16	C/T	6.22E-08	2.23E-10
rs4421378	SAMD12	8	C/A	3.97E-10	6.68E-09
rs4598240	None	8	G/A	4.94E-12	3.33E-16
rs4647907	FGFR1	8	C/T	1.56E-09	5.61E-08
rs4871593	None	8	A/G/T	3.53E-11	1.17E-08
rs521218	None	13	C/A/G	1.89E-08	1.43E-10
rs565228	TPD52L1	6	G/A	1.48E-12	5.32E-08
rs606063	None	11	T/A/C	1.73E-08	3.16E-09
rs6738783	SNRNP27	2	A/C/G	1.78E-09	3.33E-16
rs6940887	None	6	G/A	4.24E-08	2.49E-09
rs6998174	None	8	G/A	8.06E-10	9.98E-10
rs7006687	None	8	C/T	6.92E-08	4.72E-12
rs7189216	None	16	C/T	2.18E-08	1.92E-11
rs7669240	F11-AS1	4	T/C	1.21E-08	3.51E-09
rs7697959	ADGRA3	4	T/A/C/G	2.36E-08	4.69E-08
rs7783400	None	7	C/A/T	1.13E-08	2.70E-11
rs798548	AMZ1	7	T/A/C/G	5.97E-08	4.98E-08
rs859499	PLXNA2	1	G/A	3.31E-11	3.19E-08

Table S4: The significant SNPs identified by FPLS-DC and I-FPLS-DC for the right hippocampus (a).

method				FPLS-DC	I-FPLS-DC
snp	gene	CHR	Alleles	p.value	
rs10094992	MRPS28	8	T/C	1.21E-10	7.46E-09
rs10112519	MRPS28	8	C/A/G/T	9.15E-10	4.25E-08
rs10113767	MRPS28	8	C/A/T	2.14E-09	3.93E-08
rs10190247	LOC150935	2	G/A	1.93E-08	1.58E-08
rs10507913	NONE	13	C/T	1.14E-08	5.48E-08
rs1055256	EEF1AKMT2	10	A/C/G	5.39E-13	1.21E-09
rs10901808	FAM53B	10	A/C	1.31E-11	5.50E-09
rs10901809	FAM53B	10	C/T	3.90E-14	5.14E-10
rs10901812	FAM53B	10	G/A/C	2.99E-11	1.50E-10
rs10901815	NONE	10	G/A/T	3.30E-10	1.10E-09
rs10901818	EEF1AKMT2	10	G/A/C/T	2.07E-13	1.61E-09
rs11245347	FAM53B	10	G/A/C/T	9.99E-15	6.62E-09
rs11245357	EEF1AKMT2	10	A/C/G	4.78E-13	1.79E-09
rs11637659	SMAD3	15	A/C/G/T	4.59E-08	2.22E-13
rs1182171	GNA12	7	A/G/T	4.16E-12	4.32E-09
rs1182179	GNA12	7	A/G	9.60E-13	3.25E-09
rs1182182	GNA12	7	C/T	1.67E-12	2.98E-09
rs1182188	GNA12	7	T/C	1.54E-12	3.82E-09
rs11989508	MRPS28	8	T/A/C	1.27E-10	3.04E-08
rs12207402	SUPT3H	6	C/T	4.32E-08	3.84E-09
rs12430184	NONE	13	C/A/T	1.74E-08	3.26E-09
rs12694787	LINC01807	2	C/A/G	2.39E-09	4.46E-11
rs12867619	NONE	13	C/G/T	2.45E-08	2.96E-08
rs12997809	LINC01807	2	T/C	7.81E-10	3.55E-08
rs1317545	LOC150935	2	T/G	2.80E-08	9.86E-09
rs1562986	MRPS28	8	G/A/C/T	3.02E-10	7.91E-09
rs1636249	GNA12	7	T/G	4.03E-11	4.11E-15
rs1636250	GNA12	7	T/C/G	6.16E-11	2.08E-14
rs1636255	NONE	7	C/A/T	4.79E-09	5.62E-14
rs16998444	IGSF5	21	G/A	5.55E-08	1.71E-16
rs16998519	IGSF5	21	T/G	4.73E-09	2.12E-10
rs16998521	IGSF5	21	G/A	5.64E-09	4.90E-12
rs16998556	IGSF5	21	G/T	6.54E-09	2.14E-11
rs17167014	LOC107986770	7	A/G	5.17E-08	1.41E-08
rs17624662	FAM53B	10	A/C/G/T	1.71E-11	5.07E-09
rs17672387	CFAP418-AS1	8	G/A	2.18E-08	3.39E-08
rs17829311	RAD51B	14	G/A	5.60E-08	1.03E-10

Table S5: The significant SNPs identified by FPLS-DC and I-FPLS-DC for the right hippocampus (b).

method				FPLS-DC	IFPLS-DC
snp	gene	CHR	Alleles	p.value	
rs2123269	MRPS28	8	C/T	9.00E-12	1.92E-09
rs2278545	SMAD3	15	T/C/G	1.84E-08	1.21E-16
rs2457398	LOC101927040	8	C/T	2.76E-09	5.63E-08
rs2467769	LOC101927040	8	C/T	6.74E-11	9.79E-11
rs2533878	GNA12	7	G/A/C/T	1.58E-14	1.89E-10
rs3802701	FAM53B	10	C/T	4.05E-08	6.62E-09
rs4581549	SOX5	12	C/T	3.85E-08	6.59E-11
rs4740097	MRPS28	8	G/A/C/T	4.14E-10	4.28E-08
rs4896087	NONE	6	G/A	2.60E-08	6.72E-13
rs4962696	NONE	10	A/G	2.43E-10	6.44E-10
rs6936034	NONE	6	C/A/T	2.18E-08	8.73E-09
rs6988730	MRPS28	8	C/A/G/T	2.28E-10	4.49E-08
rs7015849	MRPS28	8	A/C/G/T	5.38E-11	2.53E-08
rs7079846	EEF1AKMT2	10	T/C	2.60E-10	4.63E-10
rs7099316	EEF1AKMT2	10	G/A/C/T	1.91E-13	1.44E-09
rs7783400	NONE	7	C/A/T	1.27E-08	6.52E-12
rs7898003	EEF1AKMT2	10	G/A	3.60E-13	1.34E-09
rs7926726	NONE	11	A/G	6.45E-09	8.55E-12
rs798485	GNA12	7	C/G/T	8.35E-13	1.45E-09
rs798486	GNA12	7	A/C/G	3.62E-12	4.59E-09
rs798490	GNA12	7	G/A/C	2.96E-13	3.28E-10
rs798497	GNA12	7	A/G	1.37E-11	8.76E-09
rs798548	AMZ1	7	T/A/C/G	4.01E-11	1.10E-08

Table S6: I-FPLS-DC can estimate a valuable set from each chromosome. This table depicts the results of many-to-many tests conducted on a group of genes of each valuable set related with left or right hippocampus. The column name "Left group" and "Right group means that the index of group of genes of within valuable set from 1-22 chromosome.

Left group	p.value	Right group	p.value
1	$<1.000\times10^{-16}$	1	8.130×10^{-13}
2	2.531×10^{-16}	2	$<1.000\times10^{-16}$
3	$<1.000\times10^{-16}$	3	$<1.000\times10^{-16}$
4	$<1.000\times10^{-16}$	4	$<1.000\times10^{-16}$
5	$<1.000\times10^{-16}$	5	1.221×10^{-15}
6	$<1.000\times10^{-16}$	6	1.000×10^{-16}
7	2.162×10^{-05}	7	3.874×10^{-07}
8	$<1.000\times10^{-16}$	8	1.141×10^{-07}
9	$<1.000\times10^{-16}$	9	1.743×10^{-06}
10	2.340×10^{-07}	10	3.030×10^{-10}
11	$<1.000\times10^{-16}$	11	4.086×10^{-14}
12	5.476×10^{-07}	12	7.926×10^{-11}
13	$<1.000\times10^{-16}$	13	$<1.000\times10^{-16}$
14	2.275×10^{-14}	14	$<1.000\times10^{-16}$
15	$<1.000\times10^{-16}$	15	$<1.000\times10^{-16}$
16	$<1.000\times10^{-16}$	16	2.377×10^{-07}
17	$<1.000\times10^{-16}$	17	1.745×10^{-09}
18	1.430×10^{-12}	18	$<1.000\times10^{-16}$
19	$<1.000\times10^{-16}$	19	$<1.000\times10^{-16}$
20	$<1.000\times10^{-16}$	20	1.110×10^{-16}
21	4.993×10^{-11}	21	9.676×10^{-10}
22	$<1.000\times10^{-16}$	22	$<1.000\times10^{-16}$

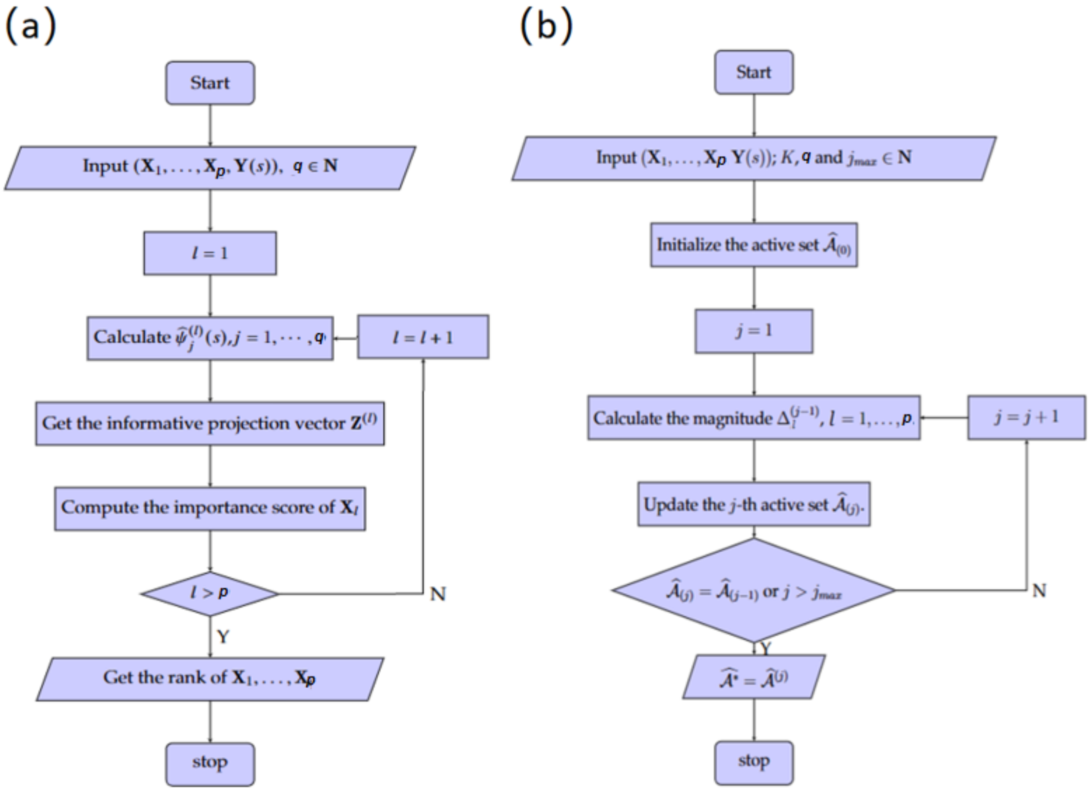

Figure S1: (a) FPLS-DC flow chart, (b) I-FPLS-DC flow chart

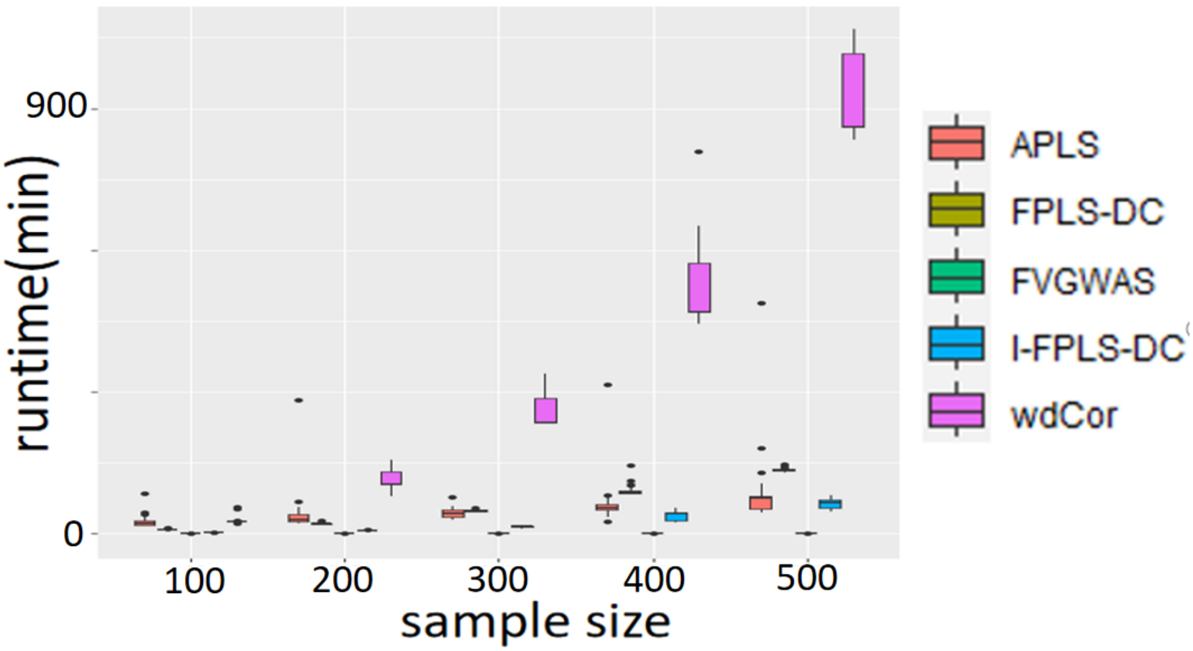

Figure S2: The runtime of FPLS-DC, I-FPLS-DC, wdCor, APLS, and FVGWAS. The sample sizes range from 100 to 500, with a predictor variable dimension of 100 and a response variable dimension of 200. Both the predictor and response variables follow a standard normal distribution.In each experiment, the computation time corresponds to the sum of the testing times for each dimension of the predictor variable.

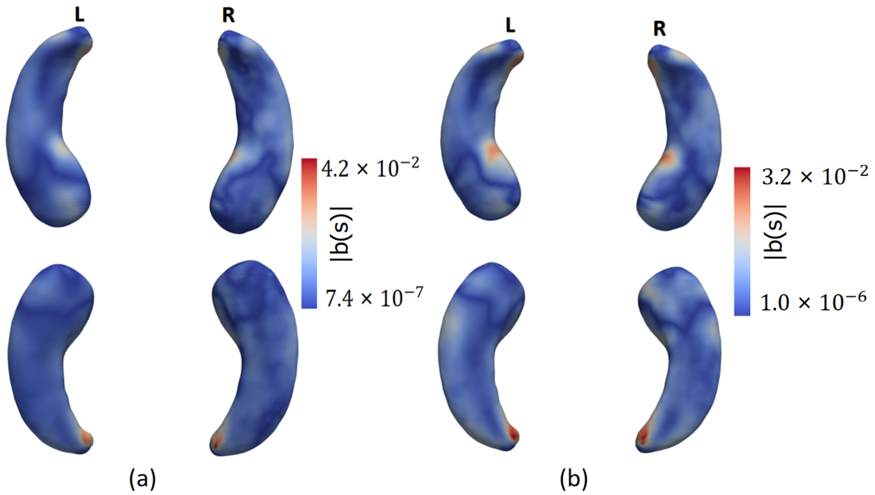

Figure S3: The heat map plots depict the impact of the most significant single nucleotide polymorphisms (SNPs) detected by FPLS-DC (a) and I-FPLS-DC (b) on the right hippocampus and left hippocampus. It is generated through variations in the absolute values of the coefficient function $b(s)$, denoted as $|b(s)|$. Larger values of $|b(s)|$ corresponding to specific subregions indicate a more substantial influence of the detected SNPs on those particular subregions.

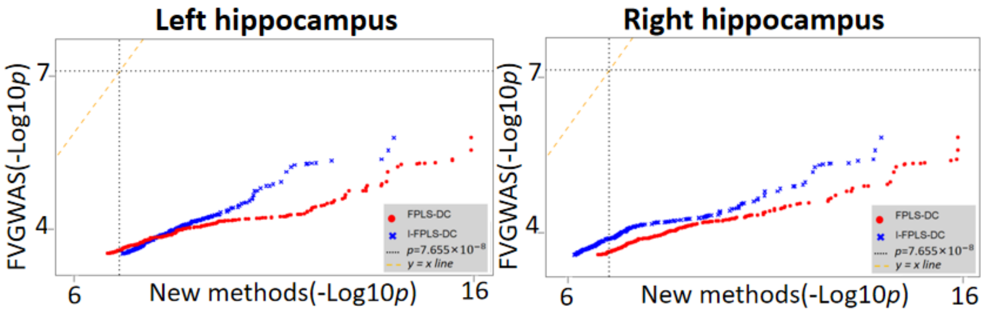

Figure S4: The -Log10p-values of top identified SNPs for proposed (FPLS-DC and I-FPLS-DC) versus existing methods (FVGWAS).

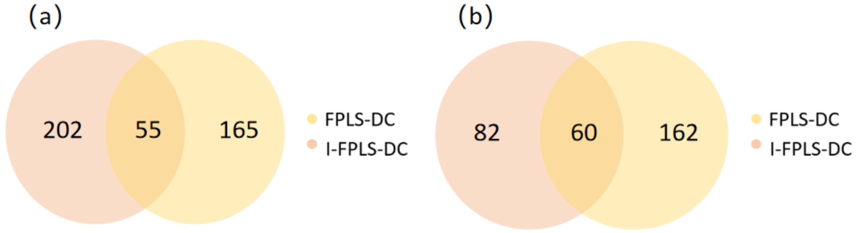

Figure S5: A Venn diagram of FPLS-DC and I-FPLS-DC on the left hippocampus (a) and right hippocampus (b).

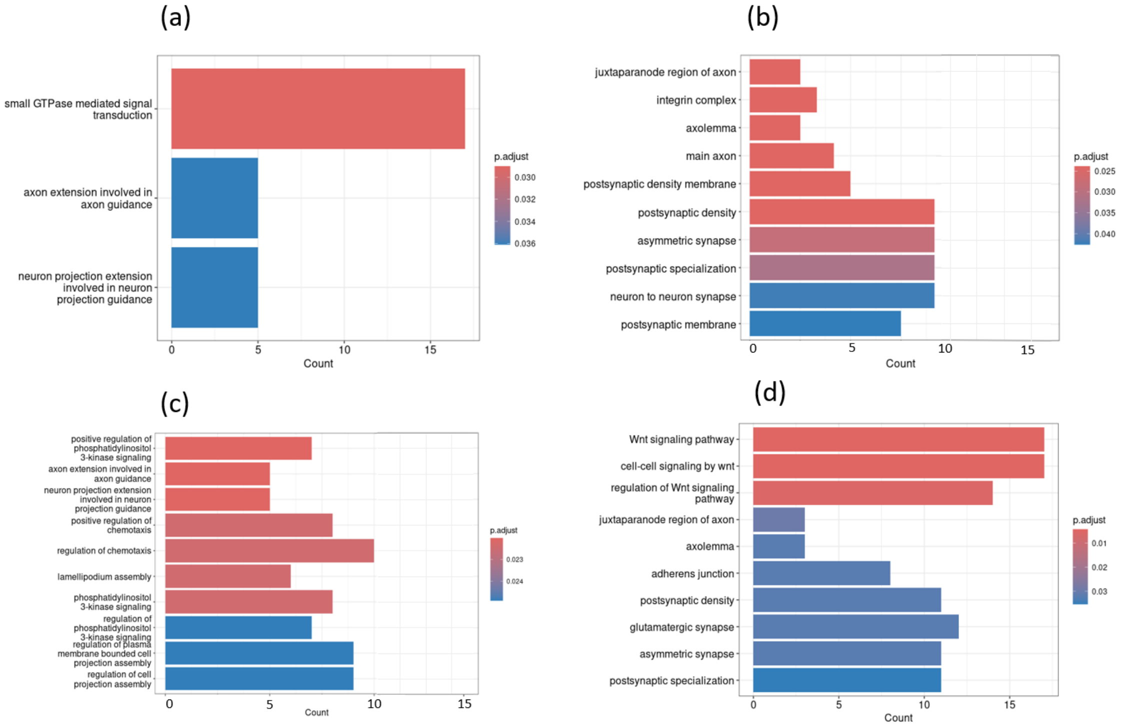

Figure S6: The color of the bars corresponds to the adjusted p-value (p.adjust) of the statistical test used to determine the significance of the gene association with the GO term. The p-value is a measure of the probability that the observed association is due to chance. The color scale shows that lower p-values (more significant associations) are colored more intensely. Each row in the plot corresponds to a different biological process, with the process names listed on the y-axis. The length of each bar (listed on the x-axis) represents the count of genes associated with each GO term. A longer bar means more genes are involved in that particular process. (a) and (b) represent I-FPLS-DC's pathway enrichment analysis results on the left and right hippocampus, respectively; (c) and (d) represent FPLS-DC's pathway enrichment analysis results on the left and right hippocampus, respectively. Typically, a p-value of 0.05 or lower is considered statistically significant, but here, the values in (a)-(d) are even lower, suggesting high significance.
